# Female Athletes and the Menstrual Cycle in Team Sports: Current State of Play and Considerations for Future Research

**DOI:** 10.3390/sports12010004

**Published:** 2023-12-21

**Authors:** Kurt Vogel, Brianna Larsen, Chris McLellan, Stephen P. Bird

**Affiliations:** 1 School of Health and Medical Sciences, University of Southern Queensland, Ipswich, QLD 4305, Australia; kurt.vogel@unisq.edu.au (K.V.); brianna.larsen@unisq.edu.au (B.L.); chris.mclellan@unisq.edu.au (C.M.); 2 Centre for Health Research, University of Southern Queensland, Ipswich, QLD 4305, Australia; 3 Lions Football Club, Richlands, QLD 4077, Australia; 4 Brisbane Tigers Rugby League Club, Coorparoo, QLD 4151, Australia; 5 Florida Panthers, Sunrise, FL 33323, USA; 6 Basketball New Zealand, Wellington 6012, New Zealand

**Keywords:** menstrual cycle, female athlete, hormones, women’s sport

## Abstract

Over the past ten years there has been a dramatic rise in female sport participation and accompanying female professional national leagues across multiple sports, yet research has not followed suit. Although there are known variations between female and male physiology, training protocols in female sport are predominantly underpinned by research undertaken in male athletes. The hormonal variability experienced by women across the menstrual cycle, as well as the menstrual cycle variability between women, may contribute to the complexity of conducting rigorous physiological studies, leading to a paucity of robust sports-specific research that can be confidently applied to female athletes. Moreover, barriers exist in female sport that potentially limit the ability to conduct research, including the lack of full-time programs and limited resources. Recently, there has been increased interest in the potential effects of fluctuations in the female sex hormones, progesterone and oestrogen, on sport performance across different phases of the menstrual cycle. However, current research evaluating the menstrual cycle and physical performance (such as strength, speed, aerobic fitness, and athletes’ perception of their performance) have shown inconsistent results. Additionally, methodological design across studies has shown little consistency, making it difficult to draw firm conclusions, which potentially prevents female athletes optimising their physical and sporting performance. It further impacts coaches and sports science researchers in their ability to provide appropriate training recommendations and educational opportunities. It is important to progress in female athlete research with an understanding of how the unique physiology of female athletes may influence their ability to physically perform in their respective sport, which requires representation in sports science research. This paper will provide an overview on current evidence and limitations within menstrual cycle research and provide considerations and directions for future research in this space within team sports.

## 1. Introduction

Over the past ten years, there has been a dramatic rise in female sport participation and accompanying female professional national leagues across Australia in sports such as football, rugby league, rugby, and cricket [1,2]. There have been similar rises globally, such as college basketball teams in the United States recently reaching parity in total teams for the first time [3], the Women’s National Basketball Association (WBNA) expansion in the United States [4], the re-introduction of Tour de France Femmes in France [5], a new Women’s Premier League Twenty20 Cricket tournament in India [6], and an entirely new professional football league in the US planned for 2024–2025 [7]. Additionally, global investment into women’s sport has grown drastically, from $481 million to $1.28 billion in only five years [8,9]. Television broadcast deals are also increasing across female sport, including a recent deal whereby nearly all Union of European Football (UEFA) Women’s Champions League matches will be streamed for free, globally, across a four-year period [10].

However, when exploring the performance literature examining female athletes and women’s sport, only 32–37% of research participants are female [11,12]. Recently, Paul and colleagues [13] evaluated over 12,000 publications to determine inequalities between male versus female athletes in sports medicine research. They identified that only 8.8% of published research in high impact sport and exercise science journals reported female-only participants, whilst 20.5% used both male and female cohorts. This disparity may be due, in part, that, historically, there have been fewer high-performance opportunities for female athletes when compared to male athletes [11,12].

Additionally, there are limitations to the availability of both public and database data, financial and promotional incentives, a higher percentage of male sports medicine clinicians and researchers, and sex biases in sport [13]. Collectively, the accuracy of female athlete data reporting may be called into question as not all studies ‘controlled’ for the menstrual cycle, a known female physiological phenomenon that is characterised by cyclical fluctuations in the female sex hormones oestrogen and progesterone. Beyond reproduction, these fluctuating sex hormones influence various physiological pathways, particularly those relevant to exercise [12]. For instance, hormones oestrogen and progesterone are known to be influential in modulating cognition, thermoregulation, substrate metabolism, and inflammation, all of which can influence exercise capacity [14,15,16,17]. The practicality of and limited funding to obtain specific physiological data such as bloods and urine across multiple phases of the menstrual cycle can make this difficult to control [18]. Where studies have controlled for the menstrual cycle, there are inconsistencies in the methodologies and the analysis of different menstrual cycle phases across the body of research. This has led to inconsistent research findings and an inability to directly compare study outcomes. The result of this research disparity is that training protocols in female sport are predominantly underpinned by research undertaken in male athlete populations [11,12]. In addition, other barriers exist within female sport that limit the ability to conduct research, such as insufficient training time due to preference of male teams, lack of resources and equipment, part-time performance staff, and limited funding [11,19]. This makes it difficult to implement appropriate, rigorous, and applied research in female sport.

As such, developing female athlete-specific physical performance protocols within research that consider and appropriately control for the menstrual cycle is a significant challenge. With such large gaps in the research, there is a need to gain a better understanding of how the menstrual cycle may affect the physical performance of female athletes. This article will explore the current state of women’s sport science research and provide recommendation guidelines for female athlete physical performance testing protocols to be used in future research.

## 2. Women’s Sport in Australia: Current State of Play

Professional female sports are continually gaining momentum and representation at the elite level. This is evidenced by Australian female athletes outnumbering male athletes for the first time at the 2016 Rio Olympic Games [20]. This trend continued at the 2020 Tokyo Olympic Games, with female athletes representing 47.8% of overall participants [21]. Despite increased level of participation, the opportunity for female athletes to pursue a full-time sporting career is limited. Most female professional league seasons only operate for three to six months, often with limited player remuneration and minimum contract term. In fact, only a few professional women’s leagues pay their players above minimum wage requirements, which presents significant liveability complications outside of the athletes’ full-time commitments. Table 1 presents the length of full-time contracts and associated contractual remuneration for professional female athletes from an Australian sporting context. Out of seven professional leagues, only one operates on annual full-time employment [22].

Within the Australian sport environment, several national programs provide full-time employment for women; however, this is not without limitations. For example, Rugby 7s is limited to major international competitions, such as the World Rugby Sevens Series and pinnacle sporting events, such as the Olympic Games. With a squad comprising of ~15 athletes, to make a national squad you must first play in a women’s competition that remunerates athletes less than half of Australia’s minimum wage for employees. In contrast, the National Rugby League men’s competition operated on a $12.1 million salary cap per team for the 2023 season [24], whilst the NRLW operated on $900,000 (eq. $3.6 million) salary cap per team for the 2023 season [25]. The Australian Bureau of Statistics reports that for women that listed themselves as a sportsperson, the average full-time wage is $42,900, whilst for their male counterparts it was $67,652—a 45% difference [26]. Additionally, facilities for female athletes often fall below the required standards. For example, Cricket Australia conducted an audit on cricket facilities around Australia and found that less than 21% of facilities are suitable for female athletes [27,28,29]. This document refers to facilities such as unisex changing rooms and toilets, adequate lighting in and around the facility, and improved safety measures. Limited facilities for female athletes is directly related to sport participation; concerns around safety in a sporting environment [30] can ultimately lead to the exclusion of women from sport [19]. Commonly, men’s sporting teams receive preference over women’s at shared facilities, which can lead to less training time, and reduced sleep (from later training starts) [30], both of which are factors that can affect training and performance. Such examples highlight some of the financial and logistical barriers that may currently limit the ability to conduct robust research in female sport. However, given the increase in women’s sport participation [1,2] and unique female physiology [31], it is imperative there is greater investment in the application of sports science research to further our understanding of how the menstrual cycle may affect physical performance.

## 3. Materials and Methods

A narrative literature review was conducted of menstrual cycle literature within sport and exercise science. The databases searched included PubMed (MEDLINE), SCOPUS and Google Scholar. All articles were selected based on a title search, year of publication (between 1990 and 2023), abstract screening, and full article screening for relevance to physical performance testing. Inclusion criteria included papers that assessed physical performance tests such as aerobic, anaerobic, strength, power, or speed tests in female athletes to determine performance differences across the menstrual cycle. Papers were excluded if participants were using hormonal contraceptives, had other known menstrual cycle irregularities, or assessed specific sporting performance. Search terms included a combination of terms: “menstrual cycle”, “physical performance”, “aerobic”, “anaerobic”, “power”, “strength”, “speed”, “athletic”, “performance”, and “testing”. Articles that did not have their full text available were not included in the final analysis. Research was included and identified based on their relevance to assessing physical performance across the different phases of the menstrual cycle.

## 4. The Menstrual Cycle

**Figure 1:**
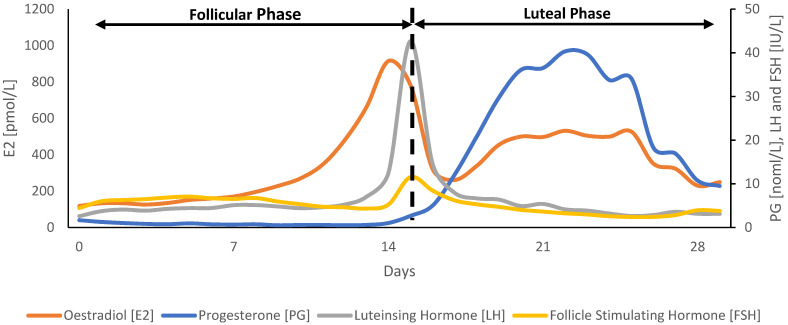
Hormonal fluctuations during a normal 30-day menstrual cycle. Adapted from Stricker et al. [34].

Menstrual cycle-related hormonal fluctuations start during puberty and occur due to an increase in luteinising hormone (LH) and follicle stimulating hormone (FSH) production. This leads to the process of ovulation (in preparation for potential pregnancy). The average menstrual cycle lasts 28 days, with evidence identifying normal variations from 21 to 35 days [32]; however, cycle length can be shorter or longer in those with menstrual disorders, such as luteal phase defects [33]. Within the cycle there are three distinct phases, characterised by different variations of the female sex hormones: the follicular phase, ovulation, and the luteal phase (Figure 1).

These phases may reflect a different hormonal profile in women who are anovulatory or have luteal phase defects [18]. Women who are anovulatory or have luteal phase defects are also commonly linked with having relative energy deficiency in sport (RED-S) [35], which can lead to increased prevalence of injuries and viral illnesses, and reduced adaptation to training and subsequent performance. This can pose much larger complications beyond performance, including low bone mineral density, increased injury risk, decreased coordination, and decreased muscle strength [35]. RED-S is especially prevalent in athletic women, where even recreational runners have been reported to have a high frequency of luteal phase defects (42%), anovulation (12%), and inconsistency between menstrual cycles (46%) [36]. The continually fluctuating hormones across the menstrual cycle may alter valid conclusions on the effects of the menstrual cycle phases on sporting performance if not appropriately accounted for.

The fluctuation of hormones during the menstrual cycle may be further confounded by the use of hormonal contraceptives. Hormonal contraceptives are a form of birth control that contain varying combinations and concentrations of synthetic oestrogen and/or progesterone to prevent pregnancy. They are prescribed in multiple forms (Table 2) that manipulate the hormonal profile of females to induce physiological changes that prevent pregnancy and affect the regular menstrual cycle [37]. Hormonal contraceptive use may also reduce exercise performance compared to regularly menstruating women; however, outcomes of research have been variable across studies [38]. For instance, hormonal contraceptive use has been shown to influence thermoregulatory processes [39], inflammation [40], and perceived exertion [39] when compared to naturally menstruating women. Due to the potential influence of hormonal contraception on physical performance, this can become another confounding variable to menstrual cycle research (particularly if hormonal contraceptive use is not recorded or accounted for in the study methodology and analysis). Due to the hormonal contraceptive effects on naturally cycling hormones [41], it can be difficult to determine whether research outcomes are due to an intervention or variability in female sex hormone concentrations. Overall, the variability of hormonal profiles across female athletes in sport (including eumenorrheic women, those with menstrual cycle disorders/dysfunction, and hormonal contraceptive users) makes it difficult to draw firm conclusions from the existing research, especially when evaluating potential effects on physical performance. For the purposes of this paper, we will be focusing on studies that have investigated potential performance differences across different stages of the menstrual cycle (in the absence of hormonal contraceptive use), with acknowledgment that further research also needs to continue to examine the role of hormonal contraception on exercise performance.

**Table 2:** Different types of hormonal contraceptives in Australia.

Type	Hormones Released	Concentration	Frequency
Oral Contraceptive Pill	Oestradiol combined with Progestogen	20–3000 micrograms 100–3000 micrograms (>30 brands, >20 different variations)	Daily
Mini Pill	Levonorgestrel Norethisterone	30 micrograms 350 micrograms	Daily
Implants	Etonogestrel (progestin)	68 mg	Replaced every 3 years
Injection	Medroxyprogesterone acetate (progestin)	150 mg/mL (single dose)	Injection every 3 months
Intravaginal Ring	Ethinyl oestradiol (oestrogen) combined with etonogesterel (progestin)	11.7 mg (0.015 mg/day) 2.7 mg (0.120 mg/day) For 3 weeks	Ring lasts three weeks, remove for one week. Replace.
Hormonal Intrauterine Device	Levonorgestrel (progestogen)	19.5 mg 52 mg (20 micrograms/24 h)	
Emergency Contraception (Pill)	Levonorgestrel (progestogen) Ulipristal acetate (progesterone receptor modulator)	1.5 mg 30 mg	Single use – emergency

Source: National Prescribing Service Medicine Wise [42,43] and Family Planning Victoria [44].

## 5. Effects of the Menstrual Cycle on Sports Performance

Research investigating sports performance is typically separated into two categories: (1) perception (subjective data) and (2) physical performance (objective data). In terms of their subjective experience, female athletes have reported predominantly negative impacts on daily life, training, and game day performance related to their menstrual cycle [45,46,47,48]. This includes experiencing symptoms such as abdominal pain, back pain, and migraines which can result in missed training sessions or altering a training session [45,46,47,48]. One study used a 36-item questionnaire and identified a minority of athletes (6.45%) that perceived the menstrual cycle to positively affect training and game day performance, highlighting the variability in individual experience [48]. A summary of menstrual-related symptoms and related athlete feedback from prior research [45,46,47] is reported in Table 3. Additionally, the lead author of this paper gathered anecdotal feedback from Australian professional female athletes (*n* = 5) regarding their experience with managing their menstrual cycle in the context of professional sports. The athletes provided a comment on the menstrual cycle education/advice offered by their sporting clubs (if any) (Table 4).

Objective physical performance is commonly determined by testing specific characteristics (i.e., strength, power, speed, and endurance). Collectively, the interaction of these qualities may be quantified during sport performance under game or match conditions. For the purposes of this paper, physical performance will relate to controlled physical performance testing. Table 5 presents a summary of the key studies investigating physical performance testing related to the menstrual cycle. Reviewing these studies collectively highlights the methodological variation between studies, including (but not limited to) the level of the athlete (e.g., regional, state or national level), the presence or absence of objective verification of menstrual cycle phase, variable testing frequency/timing across the menstrual cycle, and the type of physical characteristic tested.

For instance, 13 of the 25 studies presented in Table 5 assessed muscular strength across the menstrual cycle [49,50,51,52,53,54,55,56,57,58,59,60]. Three of the studies verified menstrual cycle via bloods [49,54,61], three via urine [55,56,58], one via saliva [50], one via oral temperature [60], and five had no menstrual cycle verification protocol [51,52,53,57,59]. Objective verification of the menstrual cycle is important as females without regular ovulatory cycles may reflect a different hormonal profile [18,62]. Of the nine studies that undertook menstrual cycle verification in some form, five found no performance differences across the different phases of the menstrual cycle [49,54,55,58], whereas four observed changes in performance in EF [50], FP [56,59], and OP [60], respectively. Though these studies verified (or attempted to verify) menstrual cycle phase, the results may be called into question for additional methodological reasons, including the frequency of testing/total menstrual cycle phases assessed, differences in participant population, and the type of maximal strength test utilised.

Across the 13 studies assessing muscular strength, different time points in the menstrual cycle were assessed, making comparisons between studies difficult. Three studies evaluated only two phases of the menstrual cycle [51,54,55], five evaluated three menstrual cycle phases [49,52,53,57,58], two evaluated four menstrual cycle phases [50,59], two studies evaluated five phases [56,60], and one study evaluated six phases [61]. There was also variability in the number of cycles assessed; ten studies assessed a single menstrual cycle [50,51,53,54,55,56,57,58,60,61], two studies assessed two complete menstrual cycles [49,59], and a single study assessed three complete menstrual cycles [52]. Performance differences were observed in the two studies that evaluated five phases of the menstrual cycle, two studies that evaluated four phases of the menstrual cycle, and one study that evaluated three phases of the menstrual cycle. However, the point in the cycle where performance differences were noted varied.

Thirteen prior studies have attempted to quantify muscular endurance changes across the menstrual cycle; however, seven different strength tests and six different exercise mediums were used across studies. These include handgrip strength [49,52,59,61], maximal isometric lower body [54,57,59,60], maximal isometric upper body [54,56,61], maximal isokinetic lower body [49,50,54], one repetition maximum (1RM) upper body [53], 1RM lower body [58], and upper body muscular endurance [51,53]. Some studies used more than one performance test. It’s important to use a consistent medium for testing as some physical performance testing protocols yield more accurate data than others [63]. Performance differences have been observed in handgrip strength [59], lower body isometric [57,59,60] and isokinetic tests [50], and an isometric upper body test [56] at various points across the menstrual cycle, depending on the study.

Finally, the participant population varies from study to study. This may be important, as trained athletes may respond differently to training protocols when compared to untrained people [64,65,66,67]. Of the 13 studies that assessed muscular strength, six of these investigated healthy females (4) [57,59,60,61] or moderately active women (2) [49,55], two investigated trained female athletes [50,54], and the remainder investigated athletes competing in a variety of sports [51,52,53,56,58]. Performance differences were observed in healthy females [59,60,61], well trained athletes [50], and rowing athletes [56].

The differences in methodologies and inconsistencies seen in the muscular strength performance literature are similar across other performance metrics, as seen in Table 5. The variability of methodologies calls into question the accuracy of the current information, and the lack of consensus across studies limits the ability to provide evidence-based recommendations to female athletes and their coaching staff. This could be doing a disservice to female athletes as they strive to achieve peak performance.

Table 6 provides a summary of the number of studies that observed changes in physical performance characteristics during specific menstrual cycle phases. Characteristics such as muscular strength show changes in performance across almost all phases of the menstrual cycle, with most studies showing no phase-based differences. This makes it challenging to provide female athletes with evidence-based recommendations as to when and how their menstrual cycle may impact performance, and how, if at all, their training should be modified. It is important to note that while one study found no direct correlation between female sex hormones and performance, the authors did find changes in performance related to psychological wellbeing, which was associated with premenstrual symptoms and menses [61]. This highlights the importance of collecting subjective data (e.g., perceived effects of the menstrual cycle) and objective data (e.g., physical performance metrics) across the research.

Inconsistency in menstrual cycle and performance study findings was highlighted in Elliot-Sale and colleagues’ [68] recently published methodological considerations for studies in sport and exercise science with women participants. The article by Elliot-Sale and colleagues [68] provides a standard of practice for conducting research with female athletes. Further research is needed to confidently establish whether there are changes in physical performance in different phases of the menstrual cycle, and if so, the timing and magnitude of those changes. To come to any consensus regarding the effect of the menstrual cycle on physical performance, future research needs to consider how testing should be conducted for different performance variables, to build a body of reliable research that can be compared to one another; ultimately, allowing readers to make more informed decisions. 

**Table 5:** Summary of physical performance testing research related to the menstrual cycle.

Study	Population	Age	MC Verified	Sample Size	Performance Test	Frequency of Test	Significant Difference
Burrows et al. [69]	Highly trained runners	18–40 yrs	LH via saliva	*n = 10*	VO2Max Treadmill	EF, LF, EL, LL; 2 cycles	≠
Cook et al. [70]	Elite and non-elite athletes	~21 yrs	No	*n = 22*	Peak Power cycle ergometer + PAP	LF, O, ML; 3 cycles	↑ 5–16% ovulation
Dam et al. [61]	Healthy women	18–35 yrs	POD, FSH, LH, PRO via bloods, LH via urine	*n = 40*	Countermovement jump Handgrip strength Biceps Iso Strength Wingate Test	EF, MF, LF, EL, ML, LL; 1 cycle	↓ 6% CMJ LL compared to MF ↓ 3% Wingate peak power EF compared to ML ↓ 2–5% Wingate average power LL
De Souza et al. [71]	Well trained runners	~29 yrs	LH via urine	*n = 8*	VO2Max Treadmill 80% Submax Treadmill	EF, ML; 1 cycle	≠
Friden et al. [49]	Moderately active women	~25.3	LH via urine, FSH, LH, E2, P-4 via bloods	*n = 10*	Handgrip strength Quad isokinetic force	EF, O, ML; 2 cycles	≠
Gordon et al. [50]	Well trained athletes	~20.7 yrs	Day of test via saliva	*n = 11*	KE peak torque isokinetic dynamometer	EF, MF, ML, LL; 1 cycle	↓ peak torque EF compared to ML and LL
Graja et al. [72]	National-level Handball athletes	~22.5 yrs	POD and PRO via bloods	*n = 10*	Repeated cycle sprint Pre- and post-cycle KE Iso strength	EF, LF, ML, LL; 1 cycle	↓ KE MVC post- in LL ↓ PP and FI in LL compared to LF
Guler [51]	Amateur volleyballers	~20 yrs	No	*n = 15*	Push up Vertical jump 30 m running sprint	EL, LL; 1 cycle	≠
Guo et al. [73]	Track and field athletes	~18.5 yrs	POD, FSH, LH, PRO via bloods	*n = 25*	500 m and 2000 m rowing sprint 100 m and 200 m running sprint	ML, LL; 1 cycle	↓ 500 m rowing, 100 m and 200 m sprint time in ML compared to MF ≠ 2000 m rowing
Kishali et al. [52]	Active basketball, volleyball, judoka	~17.3 yrs	No	*n = 40*	Vertical jump Handgrip strength 20 m sprint	EF, MF, LL; 3 cycles	≠
Julian et al. [74]	Sub-elite soccer	~18.6 yrs	No	*n = 9*	Yo-Yo IET 30 m running sprint Countermovement Jump	EF, ML; 1 cycle	↓ Yo-Yo ML compared to EF ≠ 30 m Sprint No CMJ
Kose B. [53]	Kickboxing	~21 yrs	No	*n = 10*	1RM Bench press 60% RM Bench press	EF, MF, LL; 1 cycle	≠
LeBrun et al. [54]	Trained female athletes	~27.6 yrs	POD, PRO via bloods, oral temp	*n = 16*	VO2Max Treadmill AST Treadmill Endurance Performance Isokinetic KE and KF peak torque	FP, LP; 1 cycle	≠
Middleton and Wenger [75]	Moderately active women	~24.7 yrs	No	*n = 6*	Repeated cycle sprint	FP, LP; 1 cycle	↑ Power, ↑ VO_2_ consumption in LP
Miller [55]	Moderately active women	~28.6 yrs	Oral temp, LH via urine	*n = 13*	KE Iso strength Leg press Elbow flexor Iso	MF, ML; 1 cycle	≠
Phillips et al. [56]	Rowing	~26.1 yrs	LH via urine	*n = 10*	Adductor pollicus isometric strength	3 × week; 1 cycle	10% ↑ follicular phase
Romero-Moraleda et al. [58]	Triathletes	~31.5 yrs	Yes, period tracker, tympanic temp, LH via urine	*n = 13*	1RM half squat	EF, LF, ML; 1 cycle	≠
Rodrigues [57]	Healthy females	~28 yrs	No	*n = 12*	Iso leg press	EF, LF, LL; 1 cycle	↑ force late luteal phase
Sarwar et al. [59]	Healthy females	~20.7 yrs	No	*n = 10*	KN Iso strength Handgrip strength	1 × week; 2 cycles	↑ in follicular phase
Shakhlina et al. [76]	Elite 800 m and 1500 m runners	17–24 yrs	Oral temp	*n = 13*	PWC170 Cycle 4 × 400 m runs	EF, LF, O/EL, ML, LL; 2 cycles	↑ PWC170 in MF and EL ↓ 4 × 400 m time in MF, ML
Smekal et al. [77]	Healthy females	~26.6 yrs	POD, PRO via bloods, body temp	*n = 19*	VO2Max cycle ergometer	MF, ML	≠
Tenan et al. [60]	Healthy females	~24.7 yrs	Oral temp	*n = 9*	KE Iso strength	EF, LF, O, EL, LL; 1 cycle	↓ during ovulation
Tounsi et al. [78]	High level soccer players	~21.2 yrs	PRO via bloods	*n = 11*	Five-jump test Repeat Sprint Ability Yo-Yo IET	EF, LF, LL; 1 cycle	≠
Tsampoukos et al. [79]	Highly active athletic females	~20.1 yrs	POD, PRO via bloods	*n = 14*	Repeated treadmill sprint	FP, O, LP; 1 cycle	≠
Vaiksaar et al. [80]	National (Nat) and recreational (Rec) rowers	~18.4 yrs	POD and PRO via bloods	*n = 8 (Nat)* *n = 7 (Rec)*	VO2Max rowing ergometer	MF, ML; 1 cycle	≠
Wiecek et al. [81]	Healthy females	~21 yrs	Oral temp, POD and PRO via bloods	*n = 16*	20 s cycle sprint	EF, ML; 2 cycles	≠

EF—early follicular, MF—mid follicular, LF—late follicular, O—ovulation, EL—early luteal, ML—mid luteal, LL—late Luteal, POD—oestradiol, PRO—progesterone, AST—anaerobic speed test, KE—knee extensor, KF—knee flexor, Iso—isometric, PP—peak power, FI—fatigue index, CMJ—countermovement jump and ↓—decrease, ↑—increase, ≠—no significant difference.

## 6. Considerations for Future Research

Research examining the menstrual cycle and physical performance in female athletes can be difficult to interpret. As discussed, different methodologies used to assess potential performance fluctuations across the menstrual cycle has resulted in an inconsistent body of research and limits the ability to compare one study to another. A key consideration for future research is to establish baseline data for the different phases of the menstrual cycle and the performance characteristic being tested. Additionally, further research should also capture other wellbeing data such as sleep, nutrition, and current training to establish whether there are other environmental factors influencing any performance outcomes. The remainder of this review will provide recommendations for the types of performance tests that should be used when assessing potential performance differences according to menstrual cycle phase. These recommendations factor in best-practice evidence as well as practical considerations for integrating performance testing into athlete training. Moreover, recommendations will be provided as to the ideal timing of performance testing across and within menstrual cycle phases.

Physical performance testing can be highly fatiguing, depending on the mode of exercise undertaken [82,83,84]. Thus, thought must be given as to how performance testing can be integrated into athlete training in a way that does not unduly fatigue the athletes. When assessing maximal strength, maximal isometric tests such as the isometric mid-thigh pull may be a more accurate measure of maximum strength compared to dynamic testing such as one-repetition maximum exercises [63]. Maximal isometric tests are used more commonly than 1RM testing as they have a lower risk of injury, are relatively simple to administer, have high-test reliability, and are quicker to administer than traditional 1RM testing [63]. Maximal isometric strength testing can, therefore, be completed multiple times per week, which may further enhance the accuracy of captured data due to high sensitivity. This may be especially important when the aim is to capture multiple data points across menstrual cycle phases (e.g., comparing EF to LF). Moreover, the quicker testing procedure (when compared to dynamic strength testing) means that maximal isometric strength testing can easily be integrated into regular training sessions. It should be noted that when assessing physical performance on more fatiguing tests, data may need to be collected for longer time periods to account for enough recovery between tests. More fatiguing tests can further lengthen time periods of data collection to ensure enough measurements are collected to represent all phases of the menstrual cycle across a complete cycle.

When assessing explosive strength, a countermovement test can be integrated alongside a maximal isometric test [85] when assessed with a force plate. A countermovement jump has been used to monitor sporting performance, assess inter-limb asymmetries, neuromuscular fatigue, and the effectiveness of training programs [86]. Additionally, a countermovement push up has also been used in sports such as boxing [87]. These countermovement tests are reliable, quick to implement like the maximal isometric tests, and are commonly used to assess fatigue, as they can be repeated daily.

When assessing field-based testing methodologies, there are many different test types that can be undertaken (e.g., endurance, repeat sprint ability, and anaerobic power); however, maximal speed and endurance testing are two of the most important metrics when assessing physical performance across team sports [88,89]. It has been noted that female team sport athletes achieve close to their top speed within 4–5 s, or ~30 metres [90]. Due to the eccentric contribution of the hamstrings during maximal sprinting, the further the sprint, the more fatiguing it will be [91]. However, athletes should be exposed to maximal speed training during training sessions and games to reduce their risk of injury [92,93]; therefore, maximal speed can be captured more than once per week to capture multiple data points across the menstrual cycle.

Endurance testing is similar to maximal speed testing in that it can be conducted as part of a regular block of training. Regular exposure to high intensity aerobic-based conditioning (twice per week plus match play, or thrice per week) improves cardiovascular fitness and reduces the risk of injury [94,95]. The length and intensity of the test dictates fatigue [96,97,98], and the mode of exercise will be dependent on the sport in question. However, given its regular inclusion in training protocols across most sports, this is another physical test that can be run at higher frequencies to assess potential performance differences across the menstrual cycle.

Another consideration when investigating the potential effect of the menstrual cycle on sports performance is how many menstrual cycle phases should be assessed. While there are two primary phases separated by ovulation (follicular and luteal), there are five definitive fluctuations in hormones across eumenorrheic women [34,99]. This includes the menstruation (early follicular) phase, follicular phase, ovulatory phase, luteal phase, and late luteal (or pre-menstrual) phase. As identified in Table 6, select studies report fluctuations in physical performance across different phases of the menstrual cycle; however, there is limited consistency with the number of phases being evaluated or the point at which performance changes are observed. Further research suggests more rigorous methodologies are used to assess menstrual cycle status [18,100]. This includes verification across multiple phases that can identify the true hormonal fluctuations of the menstrual cycle [18]. It is recommended that all five phases are assessed to allow for greater sensitivity when determining fluctuations in physical performance.

The final consideration to note is the length of time data collection is undertaken, which can be complicated by variable menstrual cycle length among women [99]. If two full menstrual cycles have been assessed prior to commencing testing (including menstrual cycle verification of blood samples) to assess the length of the menstrual cycle amongst study participants, then testing may be completed within one full cycle with confidence that both the follicular and luteal phases of the menstrual cycle have been captured. If menstrual cycle length has not been verified prior to testing, then testing should continue for two cycles (or longer) to ensure consistency of the menstrual cycle of participants. Following these guidelines will identify potential participants with menstrual cycle irregularities, whose hormonal profile may not be reflective of a eumenorrheic cycle.

These recommendations have been made based on sound physical performance testing principles, with practicality in mind. Limited time is required, and tests can be integrated effectively into any training environment with the appropriate funding or equipment. Using these recommendations, tests can be adapted to be sport specific and still capture the underpinning qualities of athleticism. For example, when assessing maximal speed, a basketball athlete may only need to perform a 20 m maximal sprint due to court size, whereas for a field athlete a 30 m maximal sprint may be more appropriate. This paper provides scope to assess athletic qualities, without highly fatiguing an athlete during regular training and games. It also provides recommendations for further investigating the influence of the menstrual cycle on performance, to reduce the inconsistency between studies and allow for comparison of study outcomes.

## 7. Testing Considerations for Future Research—Summary

1. Type of test: maximal isometric strength testing at strongest position of movement (e.g., isometric mid-thigh pull using force plates), a countermovement explosive test (i.e., countermovement jump using force plates), maximal speed testing up to 30 m (i.e., 30 m linear speed test using laser timing gates), and maximal aerobic testing between 3 and 10 min (i.e., 1.2 km time trial).
2. Frequency: 2–3 times per week, with at least 48 h between tests for the minimum of one full menstrual cycle *
3. Menstrual cycle phases: ideally five menstrual cycle phases should be assessed (menstruation, follicular phase, ovulation, luteal phase, and pre-menstruation).
4. Length of data collection: One full menstrual cycle if the minimum two months of consistent menstrual cycle data has been recorded prior to testing and menstrual cycle hormones are within regular values, assessed via blood sampling. *If less than two months of consistent menstrual cycle data has been recorded or for athletes with irregular menstrual cycles, then undertake testing for a minimum of two full menstrual cycles.
5. Highly fatiguing physical performance tests may impose on other aspects of training, thus require longer time periods of collection for accurate data analysis.

## 8. Conclusions

Deciphering the current body of research assessing physical performance across different phases of the menstrual cycle in female athletes is complex. Inconsistent methodologies amongst the prior literature investigating the effect of the menstrual cycle on performance include differences in population, verification of the menstrual cycle, frequency of testing, and the types of test(s) used. It is highly likely that these methodological variations have, at least in part, resulted in the varied outcomes reported in previous studies. For this reason, it is imperative that there is consistency in future research that allow more accurate data to be produced across different studies. Without any guidelines or recommendations to provide consistency in physical performance testing for menstrual cycle research in team sports, academia will continue to produce papers that cannot be compared to another, inhibiting our ability to provide meaningful information to athletes and their performance support staff. This paper provides recommendations for testing considerations for menstrual cycle research investigating physical performance testing within team sports that will allow for comparison between studies. These testing recommendations have been provided with practicality in mind; due to the limited time required to undertake the tests, they can be integrated into regular training sessions. The test recommendations provided also cover the underpinning physiological requirements for most team sports, so the tests can value-add rather than detract from training. Additionally, these testing recommendations can be adapted to suit specific sport demands. It is imperative that future research also enhances their evidence base by (a) capturing data on health and wellbeing factors such as sleep, nutrition and training intensity, and volume information; (b) including youth female athletes; (c) including females with menstrual cycle irregularities; and (d) including females who are on hormonal contraceptives. It is intended that these recommendations will provide an evidence-based framework for female athletes and performance support staff to inform training practices and support female athlete health, wellbeing, and performance.

## Figures and Tables

**Table 1:** Remuneration and employment type for professional female athletes in Australia.

Sport	Professional Female Programs in Australia		
	Employment Type	Length of Season/ Contract	Minimum Wage (AUD)
Rugby League (NRLW)	Full-time	4 months	$30,000 ($90,000 eq.)
Rugby 15s (Super W)	Full-time	3 months	$4000 ($16,000 eq.)
Cricket (WBBL)	Full-time	6 months	$26,000 ($32,000 eq.)
Football (W-league)	Full-time	6 months	$16,344 ($32,688 eq.)
Australian Rules Football (AFLW)	Full-time	6 months	$39,184 ($78,368 eq.)
Basketball (WNBL)	Full-time	6 months	$15,000 ($30,000 eq.)
Netball (Super Netball)	Full-time	12 months	$43,000
Tennis	Tournament-based	12 months	Tournament-dependent

Note: Minimum wage for employees in Australia: $45,905.60/year [23] (eq. = annual salary Australian Dollar [AUD] if contractual payments continued for 12 months).

**Table 3:** Perceptions of the menstrual cycle on daily life, training, and game day performance in female athletes.

Menstrual cycle-related symptoms	Cramps/pain, weight gain, heavy bleeding, sleep disturbance, bloating, poor temperature regulation, sick/nauseous, tiredness, low energy/lethargic, change in breast size, uncoordinated, ill/cold symptoms, bad skin, headache, fainting, dizziness, lower back pain, gastrointestinal disturbance.
Quotes on MC symptoms	“*I feel blah, I feel heavier*” “*feel a bit lousy, once it comes it’s absolutely fine*” “*Really bad cramps, kind of [in] the worst times I’ll be doubled over and be retching*” “*Slight cramps, but … they only last about an hour and they’re bearable*”
Quotes on MC impact on performance	“*at the beginning of the menstrual cycle I avoid to do tough session [sic]*” “*I don’t avoid it but do sometimes have to delay things until cramps calm down*” “*If I’m feeling rotten or low on motivation, I’ll cut the session and move training to another day, instead I will do something active but not very energy requiring. It’s all of the powerful stuff that I’ll reduce down as I’m not as strong at that time because I’m not feeling it.*” “*If anything, I have to increase it [exercise]. Helps to pass quicker by maybe a day and helps the pain*”

Source: [45,46,47].

**Table 4:** The athlete’s voice: What do elite Australian female athletes have to say?

Professional Sport	Quote
Soccer Athlete	“*I do think the menstrual cycle affects training and match day. Personally, I feel heavy and bloated around my period and I fatigue easier, which makes conditioning harder, and I struggle a bit more to play when this happens too. I have never received advice from coaches on how to modify training around my menstrual cycle either.*”
Rugby League Athlete	“*I definitely think the menstrual cycle has a negative effect on my performance. I personally dread it if I have my period for a big game and sometimes big training sessions. I’m fortunate enough to have been a part of an education program in the national team that gave a lot of information and advice around the menstrual cycle and performance, however I haven’t received any advice from coaches in regards to modifying training. It’s more self-managed, based on the information we’ve been provided.*”
Australian Rules Football Athlete	“*I definitely feel as though the menstrual cycle influences my performance on both training and match day. I often feel more lethargic, heavier and more sore when having my menstrual cycle during these times compared to if I did not have it. I have never received advise from a coach regarding menstrual cycle and physical training in my career as an athlete which extends for about a 14-year period. However, at my football club we have had a health expert talk to us each year just around what the menstrual cycle is but that’s pretty much it.*”
Hockey Athlete	“*When I was a younger athlete, I had an incredibly irregular cycle and also suffered from some quite bad acne, so it was medically recommended I tried going on the oral contraceptive pill to help with both. Before going on the OCP menstruation would be particularly impactful as I never knew when it was coming and being a younger athlete, I was very self-conscious of any mishaps that may occur, so it was a source of anxiety for me especially around tours and tournaments. Whilst on the OCP menstruation hasn’t really impacted my performance as I am able to time my faux periods to be outside of times of competition and stress. Recently I have come off OCP and it went smoothly for the first 2 months but the past month the irregular nature of the cycle has been of great stress and anxiety both for performance and personal reasons. Cycle training awareness was not discussed when I was a younger athlete and now I am settled into my routine so I have not been coached about training around the cycle much, however I have a personal understanding from my own research and studies.*”
Combat Athlete	“*I feel the impact of the menstrual cycle around my training and competition has been quite intense. For this reason, I started taking the pill. In combat sports, with weight cuts particularly, there are a lot of concerns around having my period and the pill helped me manage those. I have not had any guidance or influence by coaches as it was an avoided and untouched topic, where I would be referred to chat to other females in my life about it.*”

**Table 6:** Summary of significant differences in physical performance testing across different phases of the menstrual cycle (represented as number of studies).

	Change in Physical Performance Test (Study n)					No Change in Performance (Study n)
Performance Characteristic	Menstruation (MP, EF)	Follicular Phase (FP, MP, LF)	Ovulation (O)	Luteal Phase (EL, LP, ML)	Pre- Menstrual (PM, LL)	
Aerobic capacity		1		3		7
Repeat speed	1			1	1	2
Anaerobic power	1		1	1	1	
Muscular strength	1	1	2		1	8
Maximal speed				1		4
Muscular power					1	3

## Data Availability

No new data were created or analyzed in this study. Data sharing is not applicable to this article.
